# Cerebrospinal fluid phospho-tau T217 outperforms T181 as a biomarker for the differential diagnosis of Alzheimer’s disease and PET amyloid-positive patient identification

**DOI:** 10.1186/s13195-020-00596-4

**Published:** 2020-03-17

**Authors:** Nicolas R. Barthélemy, Randall J. Bateman, Christophe Hirtz, Philippe Marin, François Becher, Chihiro Sato, Audrey Gabelle, Sylvain Lehmann

**Affiliations:** 1 Laboratoire de Biochimie Protéomique Clinique, Plateforme de Protéomique Clinique, CHU de Montpellier, INSERM, Université de Montpellier, Montpellier, France; 2 Department of Neurology, Washington University School of Medicine, Saint-Louis, MO USA; 3 Institut de Génomique Fonctionnelle, CNRS UMR5203, INSERM U1191, Université de Montpellier, Montpellier, France; 4 Service de Pharmacologie et d’Immunoanalyse (SPI), Laboratoire d’Etude du Métabolisme des Médicaments (LEMM), CEA, INRA, Université Paris Saclay, F-91191 Gif-sur-Yvette cedex, France; 5 Memory Resources and Research Center of Montpellier, Department of Neurology, Gui de Chauliac Hospital, Montpellier, France

**Keywords:** Alzheimer’s disease, Tau proteins, Cerebrospinal fluid

## Abstract

**Background:**

Cerebrospinal fluid biomarker profiles characterized by decreased amyloid-beta peptide levels and increased total and phosphorylated tau levels at threonine 181 (pT181) are currently used to discriminate between Alzheimer’s disease and other neurodegenerative diseases. However, these changes are not entirely specific to Alzheimer’s disease, and it is noteworthy that other phosphorylated isoforms of tau, possibly more specific for the disease process, have been described in the brain parenchyma of patients. The precise detection of these isoforms in biological fluids remains however a challenge.

**Methods:**

In the present study, we used the latest quantitative mass spectrometry approach, which achieves a sensitive detection in cerebrospinal fluid biomarker of two phosphorylated tau isoforms, pT181 and pT217, and first analyzed a cohort of probable Alzheimer’s disease patients and patients with other neurological disorders, including tauopathies, and a set of cognitively normal controls. We then checked the validity of our results on a second cohort comprising cognitively normal individuals and patients with mild cognitive impairments and AD stratified in terms of their amyloid status based on PiB-PET imaging methods.

**Results:**

In the first cohort, pT217 but not pT181 differentiated between Alzheimer’s disease patients and those with other neurodegenerative diseases and control subjects much more specificity and sensitivity than pT181. T217 phosphorylation was increased by 6.0-fold in patients with Alzheimer’s disease whereas T181 phosphorylation was only increased by 1.3-fold, when compared with control subjects. These results were confirmed in the case of a second cohort, in which the pT217 cerebrospinal fluid levels marked out amyloid-positive patients with a sensitivity and a specificity of more than 90% (AUC 0.961; CI 0.874 to 0.995). The pT217 concentrations were also highly correlated with the PiB-PET values (correlation coefficient 0.72; *P* < 0.001).

**Conclusions:**

Increased cerebrospinal fluid pT217 levels, more than those of pT181, are highly specific biomarkers for detecting both the preclinical and advanced forms of Alzheimer’s disease. This finding should greatly improve the diagnosis of Alzheimer’s disease, along with the correlations found to exist between pT217 levels and PiB-PET data. It also suggests that pT217 is a promising potential target for therapeutic applications and that a link exists between amyloid and tau pathology.

## Background

There still exists no widely recognized cure for Alzheimer’s disease (AD), the leading cause of dementia worldwide [1, 2]. The diagnosis of AD and the development of suitable drug treatment are highly challenging issues, and there is still a crucial need for biomarkers which can be used to detect its early forms and distinguish it from other neurodegenerative disorders. Recent developments in cerebrospinal fluid (CSF) biochemistry [3] and brain positron emission tomography (PET) imaging [1] biomarker have yielded valuable tools in relation with amyloid-β plaques and neurofibrillary tangles (NFT), respectively. The main CSF biomarker profiles currently used for identifying AD are decreased amyloid-beta 42 (Aβ42) levels, evaluated either alone or with respect to Aβ40 [4], and increased total and phosphorylated tau levels at threonine 181 (pT181) [3–6]. This approach, based mainly on immunoassays, can be used to discriminate between AD and non-AD pathologies [7–9] [10] and to detect the AD process many years prior to the onset of cognitive symptoms and complaints [7, 11]. However, changes in the total CSF and pT181 tau levels are not entirely specific to AD. They are sometimes observed in other tauopathies, such as progressive supranuclear palsy (PSP) [8] and frontotemporal lobar degeneration (FTLD) [9], as well as dementia of other kinds, where they also reflect neurodegenerative processes. Brain studies on AD have shown the presence of many hyperphosphorylated tau sites [12–15] which might promote tau aggregation and the formation of NFT [16, 17]. These phosphorylated sites might therefore constitute alternative biomarkers of AD in addition to pT181. Finding specific means of detecting the corresponding p-tau isoforms in biological fluids for diagnostic purposes is still a challenging problem as the protein is subject to multiple post-translational modifications (acetylation, ubiquitination, methylation, truncation, etc.) at many sites along its sequence in addition to phosphorylation [18, 19]. To our knowledge, only phosphorylations of threonine 231 (pT231) and serine S199 [10, 20] have been tested for AD diagnosis in CSF using commercially available IVD immunoassay kits [21]. Whether or not the performances of methods using this biomarker are more efficient than those based on pT181 has not yet been clearly established.

Mass spectrometry-based methods are more relevant than immunoassays for assessing changes in the phosphorylation levels of specific sites independently of the total protein levels, as these methods can be used to directly quantify the phosphorylated peptides and their corresponding unmodified counterparts [13–15, 22]. In the present study, we used our latest mass spectrometry (MS) approach, which gives sensitive detection of pT181 and pT217 tau not only in brain extracts, but also in the CSF [23]. We first analyzed a cohort of probable AD patients and patients with other neurological disorders, including tauopathies, and a set of cognitively normal controls. We then checked the validity of our results on a second cohort comprising cognitively normal individuals and patients with mild cognitive impairments and AD stratified in terms of their amyloid status based on Pittsburgh compound B (PiB)-PET imaging methods. The data obtained show that CSF pT217 is a much more highly specific marker than pT181 for detecting both preclinical and advanced AD. We also established the existence of a correlation between patients’ pT217 levels and the presence of amyloidosis at an early stage in the disease.

## Methods

### Subjects and samples

The first cohort used in this study focusing on AD and its differential diagnosis originated from the Montpellier Memory Research and Resources Center. All the patients consulting the center underwent a thorough clinical examination, magnetic resonance brain imaging (MRI), and standard neuropsychometric tests including the Mini-Mental State Examination (MMSE) [24]. Laboratory tests were also performed in order to rule out the presence of dementia linked to thyroid dysregulation, metabolic syndrome, and viral diseases. The cohort included 10 patients with probable AD with high levels of evidence based on the NIA diagnostic criteria [25]. To avoid circular reasoning in this study focusing on tau, we used MRI and CSF amyloid findings rather than ELISA tau and pT181 levels to determine the pattern of the neural lesions present in AD patients. These AD patients were compared with 40 patients with non-AD diseases as follows: 8 patients with frontotemporal lobar degeneration (FTLD) with consensus criteria [26], 9 with Lewy body disease (LBD) based on the McKeith criteria [27], 6 with progressive supranuclear palsy (PSP), 1 with corticobasal degeneration (CBD), 6 with adult chronic idiopathic hydrocephalus (ACIH), 2 with mixed dementia, 2 with vascular dementia with possible AD, 2 with vascular dementia, 1 with brain metastasis, and 5 control subjects without any cognitive complaints and with normal neuropsychological profiles. In this group, lumbar punctures were performed in order to detect any acute cephalalgia or focal neurological signs so as to rule out the presence of central nervous system alterations. Participants’ demographics, clinical presentation, MRI data, and neuropsychological and biomarker profiles are presented in Table 1 and SupTable1. All the participants gave their written informed consent to participate in this study, which was approved by the Montpellier University Hospital’s regional Ethics Committee (number 2011-003926-028). CSF samples were collected in polypropylene tubes under standard conditions [28]. CSF Aβ42, total tau, and pT181 levels were measured using the standardized commercially available Innotest® sandwich ELISA (Fujirebio). The quality/accuracy of the results was ensured by using internal and external quality control (QC) procedures [29]. The QC coefficient of variation obtained on CSF total tau and pT181 in each batch and between batches ranged consistently below 15%. CSF samples were stored at the Montpellier CSF-Neurobank (#DC-2008-417 of the certified NFS 96-900 CHU resource center BB-0033-00031, www.biobanques.eu). Authorization to handle personal data was granted by the French Data Protection Authority (CNIL) under the number 1709743 v0.

**Table 1:** Demographical and cerebrospinal fluid (CSF) biomarker values in the Montpellier (AD and NAD) and the WUSTL (amyloid (−) and (+)) cohorts. Results are expressed as means ± standard deviations (SDs). *Abbreviations*: MMSE Mini-Mental State Examination, AD Alzheimer’s disease, NAD non-Alzheimer’s disease; *P* significance level of the Student’s *t* test

**Montpellier cohort**	**NAD**	***n*** **= 40**	**AD**	***n*** **= 10**	
**Variable**	**Mean**	**SD**	**Mean**	**SD**	***P***
**Age (years)**	69.3	12.1	75.7	10.1	0.1330
**Sex (% male)**	67.5%	–	20.0%	–	0.0070*
**MMSE**	21.2	6.2	18.5	4.4	0.2112
**E_Tau (pg/mL)**	260	182	715	304	< 0.0001
**E_pT181 (pg/mL)**	42.9	24.6	95.8	25.0	< 0.0001
**MS_pT181 (fmol/mL)**	23.8	13.2	60.2	27.7	< 0.0001
**MS_pT217 (fmol/mL)**	0.948	1.066	11.740	5.006	< 0.0001
**WUSTL cohort**	**Amyloid (−)**	***n*** **= 51**	**Amyloid (+)**	***n*** **= 33**	
**Variable**	**Mean**	**SD**	**Mean**	**SD**	***P***
**Age (years)**	62.7	14.3	67.0	16.3	0.2063
**Sex (% male)**	39.2%	–	57.6%	–	0.1007*
**CDR-SB**	0.34	1.09	1.77	1.82	0.0004
**%E4**	17.7%	–	66.7%	–	< 0.0001*
**PiB-PET**	0.046	0.047	0.670	0.293	< 0.0001
**E_pT181 (pg/mL)**	47.5	21.5	76.0	25.4	< 0.0001
**MS_pT181 (ng/mL)**	0.350	0.256	0.596	0.312	< 0.0001
**MS_pT217 (ng/mL)**	0.057	0.1121	0.202	0.126	< 0.0001

*Chi-squared test for the comparison of two proportions

The second cohort included amyloid-positive and prodromal AD participants, cognitively normal individuals, and patients with mild cognitive impairments recruited from the Alzheimer’s Disease Research Center (ADRC) at the Washington University in Saint Louis (WUSTL) as previously published by Patterson et al. [30]. In this ethically approved cohort, deposition of amyloid plaques was quantified in terms of the mean cortical binding potential (MCBP) the [11C]PiB-PET score (amyloid positive if the PiB-PET MCBP score > 0.1814). CSF was collected and stored as described in the latter study. Total tau and p-tau (181) CSF levels were measured using the standardized commercially available Innotest® sandwich ELISA (Fujirebio). This cohort included 33 amyloid-positive and 51 amyloid-negative participants, based on the results of the PiB-PET: the patients’ demographics of participants are described in Table 1.

### CSF tau purification and digestion

In the case of the Montpellier cohort, CSF samples (450 μL) spiked with recombinant 15N-tau-441 (final concentration 100 fmol/mL) were extracted as previously described [31]. After brief perchloric acid precipitation, acidic supernatant was extracted by performing solid phase extraction, dried, and then digested with trypsin. For the phosphorylated peptide quantification, the following synthetic heavy isotope-labeled phosphorylated peptides (AQUA, labeled at the C-terminal residue) TPPAPKpTPPSSGEPPK (pT181) and TPSLPpTPPTREPK (pT217) (Thermo Fisher Scientific, Ulm, Germany) were spiked in each sample to obtain a concentration of 100 fmol/ml. In the case of the WUSTL cohort, 800 μL of CSF supernatant obtained after Aβ immunoprecipitation [30] and stored at − 80 °C was used for tau analysis. Thawed supernatants were spiked with 15N tau internal standard (5 ng per sample, notably to control preanalytical variation) and extracted as previously described [22] by performing Tau1 immunoprecipitation. This immunoprecipitation ensured a more specific tau enrichment than chemical purification, reducing LC-MS/MS interference and ensuring a phosphopeptide signal higher by a factor 2 to 3. This could however induce a bias in p-tau detection as Tau1 antibody is sensitive to phosphorylation in its epitope. Though Tau1 failed to recover pS199, pT217 and pT181 phosphorylated isoforms are recovered by this antibody in a similar way to what is observed with some other tau antibodies [23]. Briefly, 5 mM guanidine, 1% NP-40, and protease inhibitor cocktail were added to the sample. Samples were then mixed for 3 h at room temperature with 20 μL of sepharose beads cross-linked to Tau1 antibody. Beads were precipitated before being rinsed with 0.5 M guanidine and triethylammonium bicarbonate (TEABC) 25 mM. Samples were digested with 400 ng of trypsin. In each sample, the TPPAPKpTPPSSGEPPK (pT181) and TPSLPpTPPTR (pT217) AQUA peptides were spiked along with their unphosphorylated counterparts TPPSSGEPPK (T181) and TPSLPTPPTR (T217) at 10 and 100 fmol per sample, respectively. The resulting peptide samples were loaded onto TopTip C18 tips, washed with 0.1% formic acid (FA) solution, and eluted with 60% acetonitrile (ACN) 0.1% FA solution. Samples were dried in a Speedvac and stored at − 80 °C. Prior to LC-MS analysis, samples were resuspended in 25 μL 2% ACN 0.1% FA.

### Mass spectrometry assays

In the case of the Montpellier cohort, MS analysis of endogenous and spiked peptides was performed as described elsewhere, using a LC-ESI-Quadrupole-Orbitrap analytical system (Q-Exactive, Thermo Scientific, San Jose, CA). Three microliters of each CSF digested sample was injected. Peptide separation was achieved within 30 min on a C18 column with the mobile phases (A) 0.1% formic acid in water and (B) 0.1% FA in ACN. Gradients used were 0 min-2%, 3 min-4%, 15 min-15%, 15.6 min-52%, 16 min-90%, 17.3 min-90%, 17.5 min-2%, and 30 min-2%. In the case of the WUSTL cohort, experiments were performed as previously described [22] using a Fusion Tribrid mass spectrometer (Thermo Scientific, San Jose, CA). Five microliters of each sample was injected. Peptide separation was achieved within 30 min on a Waters HSS T3 column. Mobile phases were (A) 0.1% FA in water and (B) 0.1% formic acid in ACN. Gradients used were 0 min-0.5%, 3 min-6%, 17 min-15%, 19 min-52%, 20 min-90%, 22 min-90%, 23 min-2%, and 30 min-2%. Data were acquired with both methods in the positive ion mode.

### Phosphorylated and non-phosphorylated Tau peptide quantification

Since no labeled p-tau proteins were available, AQUA phosphorylated peptides were used: this approach is generally considered to be the best source of reference material for monitoring endogenous phospho-peptide levels. In the Montpellier cohort, the absolute concentrations of unphosphorylated peptide were computed with respect to the corresponding 15N-labeled peptides, as previously described [31]. The level of each endogenous phosphorylated peptide was calculated using single point calibration methods to compare the area obtained on AQUA phosphorylated peptide counterparts. In the WUSTL cohort, pT217 and pT181 levels were calculated as follows: ptau/tau ratio for each site is calculated by comparison with AQUA internal phosphorylated and unphosphorylated peptide signals. The ptau level is then calculated by multiplying ptau/tau ratio to the t-tau level measured for the corresponding unmodified peptide using the comparison to the 15N peptide signal from recombinant tau internal standard. Tau phosphorylation on other sites, like pS214 or pS184/S185, could bias the estimation of the phosphorylation ratio at T217 or T181 respectively by contributing to the level decrease of corresponding unphosphorylated peptide. However, the phosphorylation occupancy at these sites was respectively measured as 10 to 100 times less than at positions T217 and T181 in the brain and CSF [23] which therefore reduces this potential contribution.

Measurement reproducibility was as assessed using quality controls consisting of CSF pools extracted four times independently and analyzed simultaneously within the cohort (see Sup Table 4). The response linearity of the PRM transitions used for phosphorylated peptide quantification was confirmed by the reverse curves obtained on phosphorylated AQUA peptides in pools of CSF extracts.

### Statistical analysis

Statistical analyses were performed using the MedCalc software program (19.0.5). With a non-normal distribution, the chi-squared test or Fisher’s exact test was performed on the qualitative variables and the Kruskal-Wallis test on the quantitative variables. To test the normal distribution, Student’s *t* test was used. Spearman correlations were performed. Receiver operating characteristic (ROC) curves were used to represent the sensitivity and specificity of the amyloid-positive patient detection levels. Significance level was set at *P* < 0.05.

## Results

### CSF tau and p-tau quantification on the Montpellier AD cohort

**Fig. 1:**
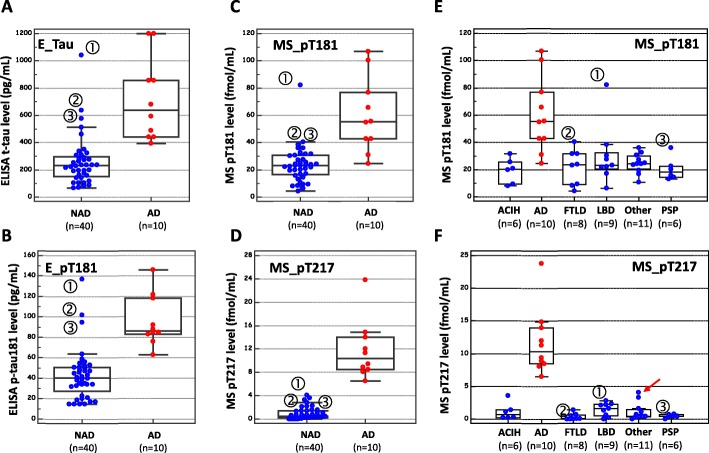
CSF tau and p-tau levels in the Montpellier AD cohort. CSF concentration of total tau (E_tau) and pT181 (E_pT181) measured by ELISA (in pg/mL) in the NAD and AD population (**a**, **b**). CSF concentration of pT181 and pT217 measured by quantitative mass spectrometry (MS) (in fmol/mL) in the NAD and AD population (**c**, **d**) and in the diseases included in the cohort (**e**, **f**). ①, ②, and ③ indicate participants with high pT181 level but normal pT217 level. The red arrow indicates participants with mixed dementia having higher MS_p217 level than controls. Differences between NAD and AD populations are statically significant (see Table 1). Abbreviations: AD Alzheimer disease, NAD non-Alzheimer disease, FTLD frontotemporal lobar degeneration, LBD Lewy body dementia, PSP progressive supranuclear palsy, ACIH adult chronic idiopathic hydrocephalus

The Montpellier cohort used in this study was composed of a relatively small number of samples collected from thoroughly characterized patients (Sup Table 1). This cohort consisted of AD patients diagnosed with a high level of confidence [25] and non-AD (NAD) patients with various other neurological disorders (PSP, LBD, FTLD) associated with cognitive deficits. These pathologies are often used for the identification of specific AD biomarkers as they represent classical differential diagnosis of AD. As expected, we observed the existence of a significant difference (*P* < 0.0001) between AD and NAD patients in terms of the ELISA values of total tau (E_Tau) and tau pT181 (E_pT181) (Table 1). However, as shown in Fig. 1a and b, several NAD patients obtained values regarded as pathological in the case of both biomarkers (above 400 ng/mL and 60 ng/mL of tau and p-tau (181), respectively [6]). Quantification of pT181 and pT217 by MS also showed the existence of significant differences between AD and NAD populations (Table 1). The distribution of the pT181values obtained by MS, which was clearly correlated with the ELISA values (Spearman’s coefficient 0.773; *P* < 0.0001), also overlapped between the AD and the NAD populations (Fig. 1c), whereas the p217 values discriminated strongly between the two populations (Fig. 1d). In order to investigate the differences observed between pT181 and pT217 more closely, we distributed these values between the various NAD etiologies (Fig. 1e, f). In some well-defined cases of FTLD, LBD, or PSP in which the pT181 values were in the AD range, the pT217 values were comparable to those measured in NAD subjects (Fig. 1f, black arrows). Apart from being associated with high total tau levels, no obvious clinical signs distinguishing these patients with high p181 levels were detected (SupTable1). We also noted that the two mixed dementia patients (Sup Table 1) had the highest pT217 levels in the NAD population (red arrow).

### CSF total-tau, p-tau correlation, and ratio in the Montpellier AD cohort

**Fig. 2:**
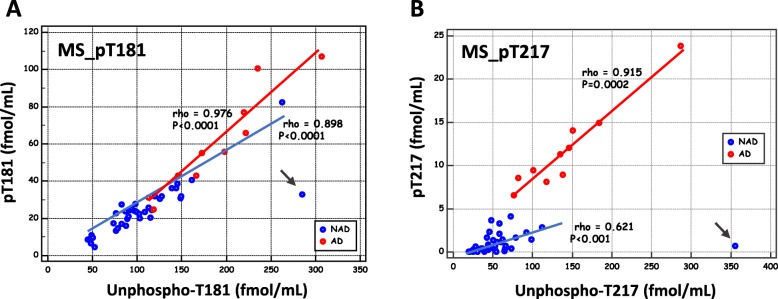
MS_pT181 and MS_pT217 plotted against their corresponding unmodified counterparts. CSF concentration of MS_pT181 (**a**) and MS_pT217 (**b**) were plotted on their corresponding unmodified counterparts in the Montpellier cohort. Linear regression was computed in the AD and NAD populations (rho Spearman’s correlation coefficient and *P* value are indicated). Note that for MS_pT217, AD and NAD regression lines have slopes that are significantly different (slope ANCOVA comparison *P* < 0.001). The sample from the patient with brain metastasis (arrow) is clearly an outliner with low concentration of phosphorylated peptides and high concentration of non-phosphorylated peptides

**Fig. 3:**
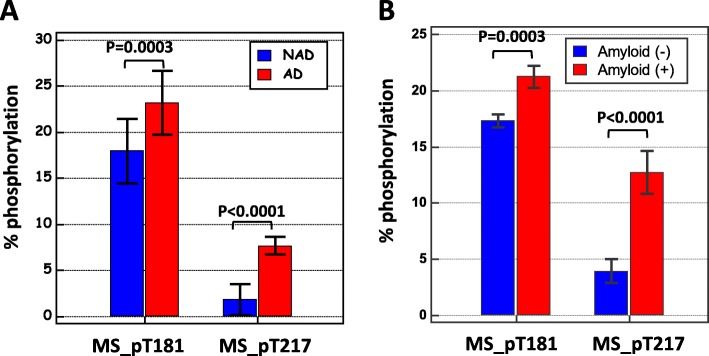
Site occupancy of phosphorylation on T181 and T217 in the two cohorts. The percentage of T181 and T217 phosphorylation corresponding to the amount of the phosphorylated peptide divided by the sum of the phosphorylated and non-phosphorylated peptide was plotted in the two cohorts. Significant differences between NAD and AD or amyloid (−) and (+) populations for the two peptides were observed (see also Sup Table 2)

In order to present the changes in tau phosphorylation state independently of the tau phosphorylation levels, pT181 and pT217 were plotted against their corresponding unmodified counterparts T181 and T217 (Fig. 2). One sample corresponding to the patient with brain metastasis (black arrow) was taken to be an outlier, since the unphosphorylated and phosphorylated levels did not follow the same variation. In addition, the correlation between the isoforms was highly significant in both NAD and AD populations (see rho Spearman’s coefficient and *P* value in the figure). Interestingly, the slopes of the correlations were higher in the AD population than in the NAD population, especially in the case of pT217 (regression formula for AD *y* = 0.426 + 0.0797*x*, for NAD *y* = 0.734 + 0.00364*x*; slope of the ANCOVA comparison *P* < 0.001). This suggests that a process of hyperphosphorylation occurs independently of the increase in tau levels in AD. To further confirm this hypothesis, we determined the p-tau to tau ratio for each phosphorylated site in the AD and NAD populations (Fig. 3a). This confirmed the occurrence of both T181 and T217 hyperphosphorylation in AD patients, corresponding to 1.3- and 6.0-fold increases in the T181 and T217 phosphorylation states, respectively, in AD vs. NAD patients (see also Sup Table 2).

### CSF tau pT181 and pT217 levels in the WUSTL cohort

**Fig. 4:**
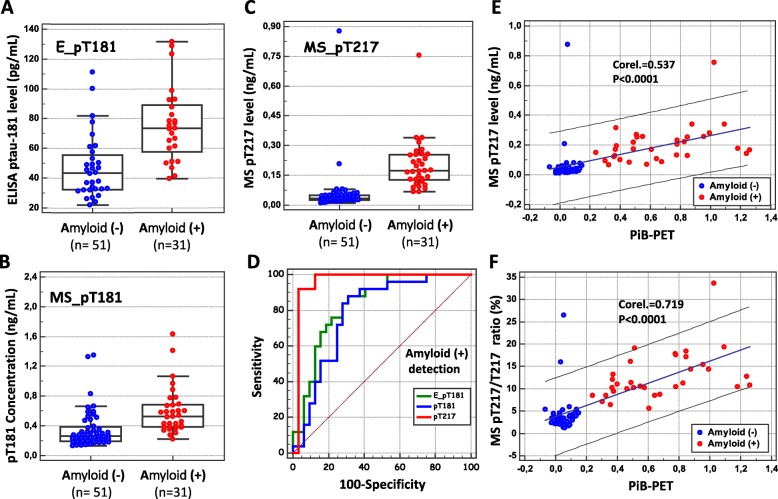
CSF p-tau in the WUSTL amyloid-positive cohort. CSF concentration of pT181 (E_pT181) was measured by ELISA (in pg/mL) (**a**), MS_pT181 (**b**), and MS_pT217 (**c**) were measured by quantitative MS (in fmol/mL) in amyloid (−) and (+) populations. The ROC curves of the detection of the amyloid (+) patients for E_pT181, MS_pT181, and MS_pT217 were plotted (**d**) (see SupTable 3 for statistical differences between curves). The MS_pT217 values (**e**) and percentage of T217 phosphorylation (% MS_pT217, **f**) were plotted against their corresponding PiB-PET values in the WUSTL cohort composed of amyloid (−) and (+) patients. Linear regression and correlation coefficients are indicated

We next investigated the levels of tau pT181 and T217 in a second cohort of patients with no cognitive complaints or only mild cognitive impairments attending the Knight ADRC at WUSTL [30, 32]. This cohort was stratified using PiB-PET in 33 amyloid-positive (+) and 51 amyloid-negative (−) participants (Table 1). To improve the sensitivity of the assay, Tau was immunoprecipitated with the Tau1 antibody (see the “Methods” and [22]). The values of pT181 measured using either ELISA or quantitative MS were found to differ significantly between amyloid (+) and (−) populations (Table 1) but overlapped significantly (Fig. 4a, b). By contrast, the pT217 also differed but overlapped less conspicuously (Fig. 4c). In order to compare the performances of these biomarkers, we computed the area under the curve (AUC) of the ROC plotting the sensitivity and specificity for amyloid (+) patient detection at different cutoffs (Fig. 4d). pT217 (AUC, 0.961) was found to be more sensitive and specific than pT181, as measured by performing either ELISA (AUC, 0.833) or MS (AUC, 0.785) (Sup Table 3). We also computed the p-tau/tau ratios of pT181 and pT217 phosphorylation (Fig. 3b) and confirmed the hyperphosphorylation of both sites in amyloid (+) patients (showing a 1.2- and a 3.5-fold increase, respectively, Sup Table 2).

### Relationship between CSF biomarkers and PiB-PET

We then examined whether there existed a correlation between amyloid PiB-PET and the total and phosphorylated tau levels in CSF from both amyloid (+) and amyloid (−) patients. Both pT181 and pT217 levels were found to be significantly correlated with the PiB-PET data, as shown in Sup Table 4 (correlation coefficients 0.418 and 0.537, respectively) and illustrated in Fig. 4e, f and in SupFigure 2. We also examined whether the p-tau/tau ratio for T217 was also correlated with the PiB-PET findings. As shown in Fig. 4f, pT217/T217 ratio was significantly correlated with the PiB-PET data, giving an even better coefficient than with the pT217 levels (correlation coefficients 0.689 and 0.719, respectively).

## Discussion

Using targeted quantitative MS to quantify low-abundance phosphorylated tau peptides (pT181 and pT217) and their unmodified counterparts, we found compelling evidence that patients’ CSF pT181 and pT217 levels had undergone AD-specific changes. These changes were characterized by an increase both in the total concentrations and in the occupancy of phosphorylation on each site measured as p-tau/tau ratios. They were particularly pronounced in the case of CSF pT217, which was 3 to 6 times more phosphorylated in preclinical to advanced AD patients from two independent, well-characterized cohorts in comparison with the corresponding control values. This finding is consistent with the increased pT217 levels detected in the brains of AD patients measured using MS methods [13, 15, 33] or with anti-p-tau antibody AT100, which recognizes pT212, pS214, and pT217 [33]. It is also in keeping with the fact that pT217 has been detected in aggregated tau extracted from AD patients’ brains [33, 34]. As far as the possible pathological consequences of T217 phosphorylation are concerned, it was previously established that preventing this process by mutating threonine 217 to alanine reduces the ability of p-tau to promote microtubule assembly in vitro [33] and to bind to SH3 domains such as the BIN1 SH3 domain [35]. All in all, these findings show that pT217 is linked to the pathophysiology of AD. The mechanism underlying T217 phosphorylation is still a matter of debate, which is also the case with the majority of tau phosphorylation sites. GSK-3, PKA, and the stress-activated protein kinases SAPK4/p38 or JNK2 generating the AT100 epitope might be involved [33, 34, 36]. Further experiments on pT217 would help to explain its high, early, specific contribution to the positive amyloid imaging results. These findings also suggest that T217 is an interesting new candidate for developing a targeted therapeutic approach in the field of AD [36]. Our results clearly show that CSF pT217 outperforms pT181 as a means of AD diagnosis. The detection of pT181 is generally held, however, to be the “gold standard” for detecting AD in the CSF [37, 38], and its recent detection in blood also suggests that this finding may lead to some interesting applications [39]. The fact that pT181 detection has been so widely used in clinical practice is no doubt due to the high levels of this peptide detected in AD patients (here it amounted to around 20% of the total tau) in comparison with other phosphorylated sites such as pT217 and to the availability of high-performance immunodetection assays which can be used for pT181 quantification. The present limitations to AD diagnosis encountered using CSF amyloid and tau biomarkers [3–6, 40–42] might be overcome if pT217 is used instead of pT181. It is our belief that the future of accurate AD diagnosis will include pT217 or other phosphorylation sites which are more specific AD markers than pT181. Universal cutoff values of pT217 optimal for AD detection will have to be determined on larger cohorts, and using reference material. To evaluate the added diagnostic value of pT217, we also compared the performance of pT181 and pT217 alone or as a ratio with Aß1-42 (SupFigure 1 and SupTable3). In the Montpellier cohort, the added value of the ratio pTau/Aß1-42 was not apparent which might be related to the poor performance of Aß1-42 in confirmed/advanced AD. In the WUSTL cohort, which focuses on amyloidosis, the added value of the ratio was present especially for pT181. Additional experiments with larger cohorts will be needed to have an optimal clinical use of the detection of pT217 alone, or most likely in combination with amyloid biomarkers. The present results also show that the pT217 levels are strongly indicative of the amyloid plaque load, as measured with PiB-PET. This finding is in line with longitudinal studies showing that amyloid impairments are involved in tau pathology [11, 43–46]. The molecular mechanism whereby amyloid and Tau pathologies contribute to AD might depend on the influence of Aβ on kinases such as GSK-3β [47, 48], which might result in the phosphorylation of tau at many sites, including T181 and pT217 [34]. Further studies focusing on T217 phosphorylation, using PET-tau imaging or other approaches, are now required in order to understand the exact role of pT217 in the cascade of molecular events that lead to AD. The finding that the pT217/T217 ratio (the percentage occupancy of phosphorylation on T217) is highly correlated with the amyloid load suggests the occurrence of a series of changes that could be used to follow the progression of the disease and/or improve the possibility of predicting the risk of cognitive decline in individuals who have converted/are converting to clinical AD [44].

### Limitations

This study has several limitations. First, the present AD cohort was rather small, which is nevertheless counterbalanced by the detailed clinical and biological data on the patients. In addition, the second cohort was stratified based on the presence of brain amyloidosis, which is not always equivalent to AD. Lastly, this study does not include any longitudinal data that might have served to document the changes in biomarkers with respect to the progression of the disease.

## Conclusions

In conclusion, the results of this study suggest that pT217 is a more accurate biomarker than pT181, which could be used to improve the diagnosis and follow-up of preclinical to advanced cases of Alzheimer’s disease. It should therefore constitute a promising new target for therapeutic applications. These findings also suggest/show that amyloid plaques contribute to the processes responsible for tau pathophysiology.

## Supplementary information

**Additional file 1: SupFigure 1.** ROC curves of biomarker combinations. The ROC curves for the detection of the amyloid (+) patients in the WUSTL cohort (Panel A) or the AD patients in the Montpellier cohort (Panel B) for Aβ1–42, MS_pT181, MS_pT217, MS_pT181/ Aβ1–42 and MS_pT217/ Aβ1–42 were plotted. AUC values and statistical differences between curves of the panel A are reported in SupTable 3.** SupFigure 2.** CSF p-tau181 correlation with Pib-PET. The MS_pT181 values (panel A) and % of T181 phosphorylation (% MS_pT181, panel B) were plotted against their corresponding PiB-PET values in the WUSTL cohort composed of amyloid (−) and (+) patients. Correlation coefficients are indicated.

**Additional file 2: SupTable 1.** Clinical and MRI characteristics of the Montpellier cohort. **SupTable 2.** Percentage of T181 and T217 phosphorylation. **SupTable 3.** AUC of phosphorylation of the different sites. **SupTable 4.** Correlation between CSF biomarkers and PiB-PET. **SupTable 5.** Internal quality control and reproducibility of the two methods.

## Data Availability

The datasets used for the analyses are available from the corresponding author on reasonable request.

## Abbreviations

ACIH: Adult chronic idiopathic hydrocephalus
ACN: Acetonitrile
AD: Alzheimer’s disease
ADRC: Alzheimer’s Disease Research Center
AQUA: Absolute quantitation
AUC: Area under the curve
Aβ: Amyloid β
CBD: Corticobasal degeneration
CI: Confidence interval
CSF: Cerebrospinal fluid
FA: Formic acid
FTLD: Frontotemporal lobar degeneration
LBD: Lewy body dementia
LC-ESI: Liquid chromatography electrospray ionization
MCBP: Mean cortical binding potential
MCI: Mild cognitive impairment
MMSE: Mini-Mental State Examination
MRI: Magnetic resonance brain imaging
NAD: Non-Alzheimer disease
NFT: Neurofibrillary tangles
PET: Positron emission tomography
PiB: Pittsburgh compound B
PSP: Progressive supranuclear palsy
QC: Quality control
ROC: Receiver operating characteristic
SD: Standard deviation
TEABC: Triethylammonium bicarbonate
WUSTL: Washington University in Saint Louis
