# Supplementary figures and images for: Cerebrospinal fluid phospho-tau T217 outperforms T181 as a biomarker for the differential diagnosis of Alzheimer’s disease and PET amyloid-positive patient identification
Source: Alzheimers Res Ther. 2020 Mar 17;12:26. doi: 10.1186/s13195-020-00596-4 (PMC7079453; doi:10.1186/s13195-020-00596-4)

SupFigure 1

A

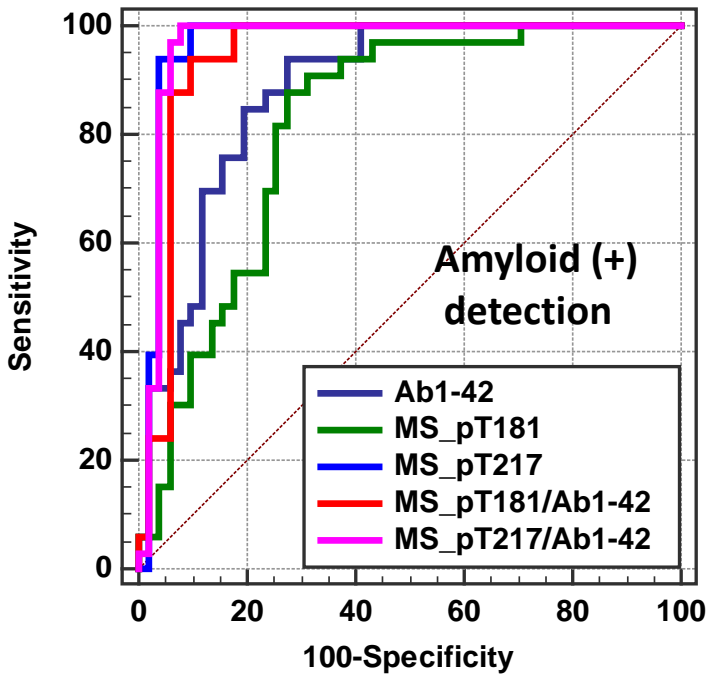

B

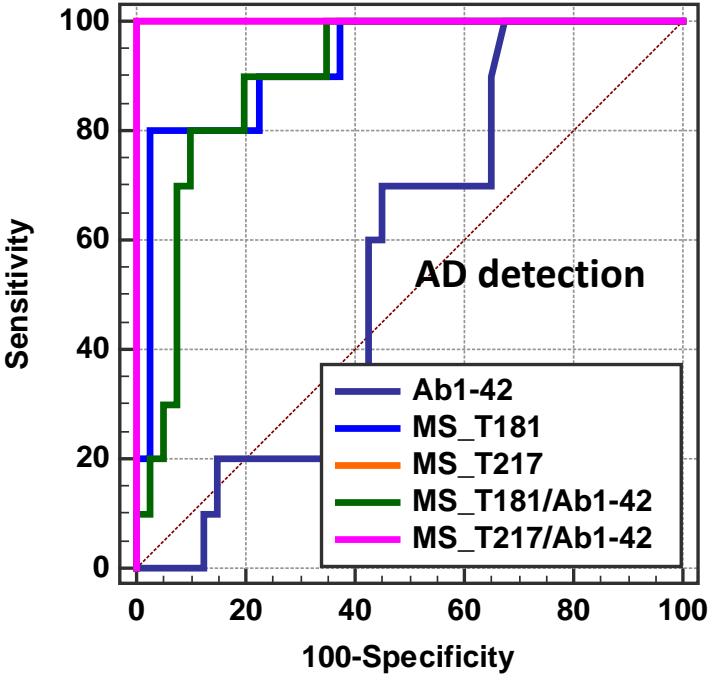

SupFigure 2

A

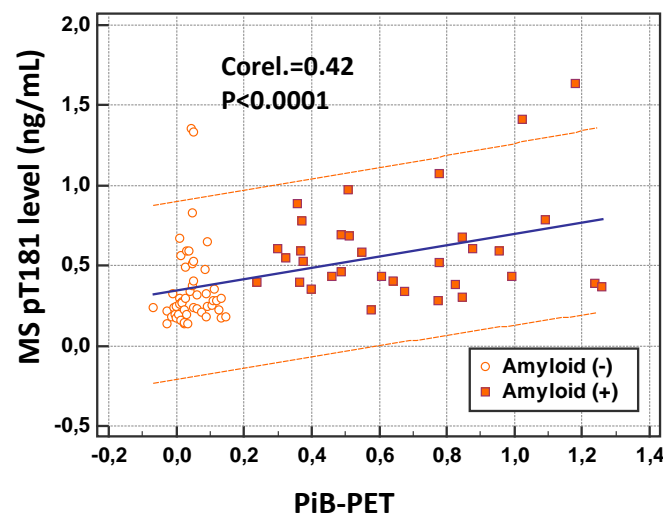

B

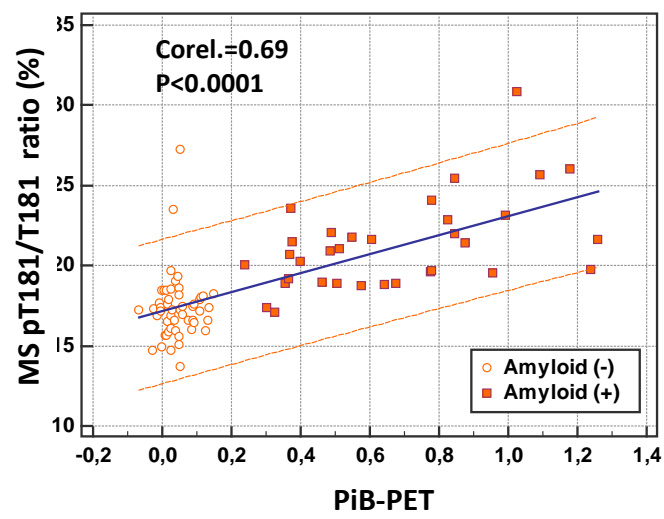

Supplement: Supplementary file 1 — Additional file 1: SupFigure 1. ROC curves of biomarker combinations. The ROC curves for the detection of the amyloid (+) patients in the WUSTL cohort (Panel A) or the AD patients in the Montpellier cohort (Panel B) for Aβ1–42, MS_pT181, MS_pT217, MS_pT181/ Aβ1–42 and MS_pT217/ Aβ1–42 were plotted. AUC values and statistical differences between curves of the panel A are reported in SupTable 3. SupFigure 2. CSF p-tau181 correlation with Pib-PET. The MS_pT181 values (panel A) and % of T181 phosphorylation (% MS_pT181, panel B) were plotted against their corresponding PiB-PET values in the WUSTL cohort composed of amyloid (−) and (+) patients. Correlation coefficients are indicated. [file 13195_2020_596_MOESM1_ESM.pdf]
