# Supplementary material for: Cerebrospinal fluid phospho-tau T217 outperforms T181 as a biomarker for the differential diagnosis of Alzheimer’s disease and PET amyloid-positive patient identification
Source: Alzheimers Res Ther. 2020 Mar 17;12:26. doi: 10.1186/s13195-020-00596-4 (PMC7079453; doi:10.1186/s13195-020-00596-4)
Supplement: Supplementary file 2 — Additional file 2: SupTable 1. Clinical and MRI characteristics of the Montpellier cohort. SupTable 2. Percentage of T181 and T217 phosphorylation. SupTable 3. AUC of phosphorylation of the different sites. SupTable 4. Correlation between CSF biomarkers and PiB-PET. SupTable 5. Internal quality control and reproducibility of the two methods. [file 13195_2020_596_MOESM2_ESM.docx]

# Sup-Tables

## SupTable 1: Clinical and MRI characteristics of the Montpellier cohort.

Abbreviations: MMSE = Mini-Mental State Examination; MRI = Magnetic Resonance Imaging; Hippocampal memory tb. = Hippocampal memory troubles related to episodic memory deficit; Behavioral tb. = Behavioral troubles (including apathy and/or disinhibition); other cognitive tb. = other cognitive troubles or decline; Fasekas = Score that evaluates the vascular load from 0 (no vascular brain lesions) to 4 (large confluent periventricular hyperintensities); HBP = High Blood Pressure; Dyslipid. = Dyslipidemia (increase of cholesterol level or triglycerides and/or dyslipidemia drugs intake); MD = Mellitus Diabetes; CarD = Cardiological disease; TIE = transient ischemic event; Semantic memory tb. = semantic memory troubles; walking prob. = Walking problems; EP signs = extrapyramidal signs (akinesia and/or rigidity and/or tremor); Pyr. Signs = Pyramidal signs (spasticity and/or hyperreflexia and/or Babinski’s signs); hallu.= visual hallucinations; RBD = REM sleep behavioral disorder; AD = Alzheimer’s disease; Mixed dementia (MD) AD with CVD = Alzheimer’s disease with cerebrovascular disease; FTLD = Frontotemporal lobar degeneration; LBD = Lewy Body dementia; PSP = Progressive Supra-nuclear Palsy; CBD = Cortico-basal Degeneration; ACIH = Adult Chronic Idiopathic Hydrocephalus; ND = Not determined.

| **Sample ID** | **Age** | **Sex** | **MMS** | **Diagnosis** | **Clinical assessment** | **Vascular Risk Factors** | **Hippocampal memory tb.** | **Dysexecutive syndrome** | **Behavioural tb.** | **Semantic memory tb., anomia, apraxia** | **MRI Atrophy** | **MRI Fasekas** |
| --- | --- | --- | --- | --- | --- | --- | --- | --- | --- | --- | --- | --- |
| **36** | 64 | M | 29 | Other (Ctrl) | Normal | None | 0 | 1 | 1 | Anomia, visual agnosia | Normal | 0 |
| **62** | 57 | F | 20 | PSP | Frontal signs, EP signs, falls | None | 0 | 1 | 1 | None | Minor global | 0 |
| **77** | 75 | M | 25 | PSP | Walking prob., EP signs, falls, depression | Stroke (TIE), CarD | 1 | 1 | 1 | Anomia | Global | 0 |
| **78** | 83 | F | 12 | LBD | Walking prob., EP signs, falls, hallu., RBD, myoclonia | HBP, Dyslipid., CarD | 0 | 1 | 0 | Anomia, apraxia, visual agnosia, cognitive fluctuations | Global | 2 |
| **82** | 79 | F | 21 | LBD | Gait tb., EP signs, hallu. | HBP, Dyslipid. | 0 | 1 | 0 | Apraxia, visual agnosia, cognitive fluctuations | Global | 0 |
| **101** | 50 | F | 30 | Other (Ctrl) | Normal | HBP, Dyslipid., Tobacco | 0 | 1 | 0 | None | Normal | 0 |
| **108** | 70 | F | 27 | LBD | Walking prob., falls | HBP | 0 | 1 | 0 | Anomia | Normal | 0 |
| **120** | 25 | F | 30 | Other (Ctrl) | Normal | Tobacco | 0 | 0 | 0 | None | Normal | 0 |
| **122** | 55 | F | 20 | LBD | Gait tb., oculomotor palsy, EP signs | None | 0 | 1 | 1 | Anomia, agnosia | Global | 1 |
| **134** | 80 | M | 3 | LBD | EP signs, frontal signs, vigilance deficit, confusion | Dyslipid., MD, CarD | 1 | 0 | 1 | Semantic memory tb., anomia, agnosia, cognitive fluctuations | Global | 1 |
| **179** | 74 | M | 22 | LBD | EP signs, hallu. | None | 1 | 1 | 0 | Semantic memory tb., anomia, apraxia, visual agnosia, cognitive fluctuations | Global | 0 |
| **184** | 75 | F | 22 | LBD | EP signs, hallu. | HBP, CarD | 1 | 1 | 0 | Semantic memory tb., apraxia, agnosia | Minor global | 0 |
| **219** | 83 | M | 17 | PSP | Walking prob., EP signs, frontal signs | HBP, Stroke, CarD, Tobacco | 0 | 1 | 0 | Anomia, apraxia | Global | 1 |
| **221** | 68 | M | 25 | Other (VD) | EP signs, depression | HBP, MD, CarD, Tobacco | 0 | 1 | 0 | Anomia, apraxia, agnosia | Minor global | 0 |
| **237** | 67 | M | 10 | LBD | EP signs, hallu., RBD | None | 0 | 1 | 1 | Anomia, agnosia | Global | 0 |
| **266** | 66 | F | NA | Other (BM) | Normal | None | 0 | 0 | 0 | Confusion | Metastasis | 0 |
| **337** | 75 | F | 25 | FTLD | Walking prob. | None | 0 | 1 | 1 | Anomia | Fronto- temporal | 0 |
| **341** | 59 | M | 17 | LBD | EP signs, hallu., depression | Tobacco | 1 | 1 | 1 | None | Global | 2 |
| **360** | 78 | M | 22 | Other (VD) | Walking prob., Pyr. Signs | Dyslipid., Stroke, MD | 0 | 1 | 0 | None | Global | 4 |
| **375** | 79 | M | 16 | ACIH | Gait & Walking prob., falls, frontal signs | HBP, Dyslipid., MD | 1 | 1 | 1 | Anomia | 0,0 | 0 |
| **406** | 78 | M | 11 | ACIH | Walking prob. | HBP, MD | 0 | 1 | 0 | Anomia, agnosia | 0,0 | 0 |
| **416** | 68 | M | 18 | FTLD | Frontal signs | HBP | 0 | 1 | 1 | Anomia | Fronto- temporal | 0 |
| **437** | 76 | F | 20 | AD | AD | Stroke, Tobacco | 0 | 1 | 1 | Anomia, apraxia, agnosia | Global | 3 |
| **443** | 56 | M | 26 | FTLD | Walking prob. | None | 0 | 1 | 0 | Anomia | Fronto- temporal | 0 |
| **449** | 84 | F | 18 | AD | AD | CarD | 1 | 1 | 1 | Anomia, apraxia, agnosia | Global | 0 |
| **459** | 62 | F | 28 | Other (Ctrl) | Normal | None | 0 | 1 | 0 | None | Normal | 0 |
| **462** | 68 | M | 25 | FTLD | Normal | None | 0 | 1 | 1 | None | Fronto- temporal | 0 |
| **504** | 81 | M | 19 | ACIH | Walking prob. | HBP, MD, CarD | 0 | 1 | 0 | Semantic memory tb., anomia, apraxia | 0,0 | 0 |
| **535** | 70 | M | 20 | ACIH | Walking prob., frontal signs | None | 0 | 1 | 1 | Anomia | 0,0 | 0 |
| **681** | 72 | M | 23 | PSP | Walking prob., EP signs, frontal signs | HBP, Dyslipid., MD | 1 | 1 | 0 | Anomia, apraxia | Frontal | 0 |
| **705** | 70 | M | 27 | FTLD | Walking prob. | None | 0 | 1 | 1 | None | 0,0 | 0 |
| **743** | 67 | M | 22 | PSP | EP signs, oculomotor palsy | None | 1 | 1 | 1 | Anomia | Global | 0 |
| **746** | 70 | M | 27 | FTLD | Walking prob. | None | 0 | 1 | 1 | None | 0,0 | 0 |
| **867** | 62 | M | 23 | FTLD | Frontal signs | Tobacco | 0 | 1 | 1 | Anomia, visual agnosia | Fronto- temporal | 0 |
| **873** | 54 | F | 14 | AD | AD | None | 1 | 1 | 1 | Semantic memory tb., anomia, apraxia | Global | 0 |
| **915** | 72 | M | 24 | ACIH | Gait & Walking prob. | HBP, Dyslipid., Stroke, MD, CarD | 1 | 1 | 1 | Anomia, Apraxia | 0,0 | 0 |
| **929** | 83 | M | 23 | AD | AD | MD | 0 | 1 | 1 | Semantic memory tb., anomia, agnosia | Global | 2 |
| **951** | 53 | M | 21 | PSP | Walk & gait tb., EP signs, oculomotor palsy | None | 0 | 1 | 1 | Anomia | Normal | 0 |
| **1022** | 90 | M | 23 | Other (MD) | Walking prob. | CarD | 1 | 1 | 0 | Semantic memory tb., anomia, apraxia, agnosia | Global | 3 |
| **1040** | 83 | F | 13 | AD | AD | None | 1 | 1 | 1 | Anomia, apraxia, agnosia | Global | 1 |
| **1040** | 83 | F | 13 | AD | AD | None | 1 | 1 | 1 | Anomia | Global | 1 |
| **1071** | 78 | F | 16 | AD | AD | Dyslipid. | 1 | 0 | 1 | Anomia | Global | 2 |
| **1123** | 54 | M | 28 | Other (Ctrl) | Walking prob., Pyr. Signs | None | 0 | 1 | 0 | None | Normal | 0 |
| **1153** | 70 | F | 21 | AD | AD | HBP, Dyslipid. | 1 | 1 | 1 | Anomia, apraxia, agnosia | Global | 0 |
| **1295** | 64 | M | 22 | AD | AD | HBP | 1 | 0 | 1 | Anomia, apraxia | Global | 0 |
| **1397** | 73 | M | 16 | Other (MD) | Gait & Walking prob., EP signs | HBP, Tobacco | 0 | 1 | 0 | Anomia | Global | 4 |
| **1403** | 83 | M | 27 | ACIH | Walking prob. | None | 0 | 1 | 1 | None | 0,0 | 0 |
| **1457** | 77 | F | 11 | FTLD | Frontal signs | CarD | 1 | 0 | 1 | None | Hippocampal | 2 |
| **1528** | 82 | F | 25 | AD | AD | HBP, CarD, Tobacco | 1 | 0 | 0 | Semantic memory tb., anomia, agnosia | Hippocampal | 0 |
| **1631** | 87 | F | 13 | Other (CBD) | Walking prob., EP signs, frontal signs | Dyslipid. | 0 | 1 | 1 | Asymetric apraxia | Parietal | 0 |

## SupTable 2: Percentage of T181 and T217 phosphorylation

The percentage of T181 and T217 phosphorylation corresponding to the amount of the phospho-peptide divided by the sum of the phospho- and non-phosphorylated peptide was calculated in the two cohorts. Results are expressed as the median/average rank for the Montpellier cohort or the mean ± standard deviation (SD) based on the normality of the distribution. P significance level of the Student’s t-test (WUSTL) and the Kruskal-Wallis test (Montpellier).

| **Montpellier cohort** | **NAD** | **n=40** | **AD** | **n=10** |  |  |
| --- | --- | --- | --- | --- | --- | --- |
| **Variable** | **Median** | **Av. range** | **Median** | **Av. range** | **P** | **fold increase** |
| **% MS_pT181** | 18.11 | 21.23 | 22.71 | 39.70 | 0.0003 | 1.3 |
| **% MS_pT217** | 1.29 | 17.56 | 7.69 | 39.30 | <0.0001 | 6.0 |
| **WUSTL cohort** | **Amyloid (-)** | **n=65** | **Amyloid (+)** | **n=47** |  |  |
| **Variable** | **Mean** | **SD** | **Mean** | **SD** | **P** | **fold increase** |
| **% MS_pT181** | 17.29 | 2.04 | 21.48 | 2.54 | <0.0001 | 1.2 |
| **% MS_pT217** | 3.77 | 3.41 | 13.22 | 5.20 | <0.0001 | 3.5 |

## SupTable 3: AUC of phosphorylation of the different sites

Area under the curve (AUC) of the ROC representation of the sensitivity and specificity for amyloid (+) patient detection were computed. Differences between AUC were tested.

| **WUSTL cohort** |  |  | **Pairwise comparison (P values)** | | | | |
| --- | --- | --- | --- | --- | --- | --- | --- |
| **Amyloid (+)** | **AUC** | **95% CI** | **E_pT181** | **pT181** | **pT217** | **Aβ1-42** | **pT181 / Aβ1-42** |
| **E_pT181** | 0.833 | 0.710 to 0.918 | / | / | / | / | / |
| **MS_pT181** | 0.785 | 0.656 to 0.883 | 0.3670 | / | / | / | / |
| **MS_pT217** | 0.961 | 0.874 to 0.995 | **0.0393** | **0.0015** | / | / | / |
| **Aβ1-42** | 0.882 | 0.793 to 0.942 | 0.5029 | 0.2961 | **0.0201** | / | / |
| **MS_pT181 / Aβ1-42** | 0.942 | 0.869 to 0.981 | 0.3670 | **0.0006** | 0.1421 | 0.1160 | / |
| **MS_pT217 / Aβ1-42** | 0.965 | 0.900 to 0.993 | **0.0393** | **0.0005** | 1.0000 | **0.0157** | 0.1680 |

## SupTable 4: Correlation between CSF biomarkers and PiB-PET

CSF concentration and percentage of phosphorylation using Spearman's correlation with PiB-PET in the WUST cohort.

| **PiB-PET** | **Correlation**  **coefficient** | **Significance**  **Level P** | **PiB-PET** | **Correlation**  **coefficient** | **Significance**  **Level P** |
| --- | --- | --- | --- | --- | --- |
| **MS_pT181** | 0.418 | 0.0001 | **% p181** | 0.689 | <0.0001 |
| **MS_pT217** | 0.537 | <0.0001 | **% pT217** | 0.719 | <0.0001 |

## SupTable 5: Internal quality control and reproducibility of the two methods

To evaluate the reproducibility of the two mass spectrometry methods we used pools of CSF with Low, Intermediate and High tau concentration. Coefficient of variation (CV) are indicated in %.

| **Tau CSF pools** | **MS_pT181 (Montpellier)** | **MS_pT181 (WUSTL)** | **MS_pT217 (Montpellier)** | **MS_pT217 (WUSTL)** |
| --- | --- | --- | --- | --- |
| **Low** | 20.5% | 23.6% | 64.8% | 18.8% |
| **Intermediate** | 11.9% | / | 35.5% | / |
| **High** | 7.8% | 26.0% | 16.2% | 14.2% |
